# Stochastic establishment of β-lactam-resistant *Escherichia coli* mutants reveals conditions for collective resistance

**DOI:** 10.1098/rspb.2021.2486

**Published:** 2022-05-11

**Authors:** Manja Saebelfeld, Suman G. Das, Arno Hagenbeek, Joachim Krug, J. Arjan G. M. de Visser

**Affiliations:** ^1^ Institute for Biological Physics, University of Cologne, Cologne, Germany; ^2^ Laboratory of Genetics, Wageningen University, Wageningen, The Netherlands

**Keywords:** establishment probability, antibiotic resistance evolution, minimum inhibitory concentration, social interactions

## Abstract

For antibiotic resistance to arise, new resistant mutants must establish in a bacterial population before they can spread via natural selection. Comprehending the stochastic factors that influence mutant establishment is crucial for a quantitative understanding of antibiotic resistance emergence. Here, we quantify the single-cell establishment probability of four *Escherichia coli* strains expressing β-lactamase alleles with different activity against the antibiotic cefotaxime, as a function of antibiotic concentration in both unstructured (liquid) and structured (agar) environments. We show that concentrations well below the minimum inhibitory concentration (MIC) can substantially hamper establishment, particularly for highly resistant mutants. While the pattern of establishment suppression is comparable in both tested environments, we find greater variability in establishment probability on agar. Using a simple branching model, we investigate possible sources of this stochasticity, including environment-dependent lineage variability, but cannot reject other possible causes. Lastly, we use the single-cell establishment probability to predict each strain's MIC in the absence of social interactions. We observe substantially higher measured than predicted MIC values, particularly for highly resistant strains, which indicates cooperative effects among resistant cells at large cell numbers, such as in standard MIC assays.

## 1. Introduction

A classical question in population genetics addresses the fate of a new adaptive allele in a population. Any new allele is initially present at a low frequency in the population and thus prone to extinction by genetic drift, even if the allele is beneficial [1,2]. In 1927, Haldane stated that a novel beneficial allele has to overcome drift loss and *establish* in the population before it can be picked up by selection; the probability of which he estimated to be roughly two times its selective benefit [3]. Since then, Haldane's approximation has been generalized and extended by a great number of models, but none of them has been tested empirically until about a decade ago [1]. With advances in genomic techniques, the issue of fixation probability has more recently been studied in experimental populations of microorganisms (e.g. [3,4]). These studies looked at the fate of beneficial alleles once they reach a relatively high frequency in the population, finding that their fixation strongly depends on the competition with further beneficial alleles that appear via mutation over time. Only a small number of empirical studies has investigated the process of establishment of an initially rare beneficial allele arisen by *de novo* mutation in fungi [6], bacteria [7,8] and nematodes [9]. Overall, these studies found that the establishment process is stochastic and that the probability for a beneficial allele to establish in a population depends on the alleles' initial frequency, its selective benefit (e.g. in response to the concentration of the selective agent), and the size of the population.

Whether a beneficial allele establishes successfully in a population is of particular relevance in cases of unwanted evolution, such as the evolution of antibiotic resistance. The worldwide growing concern about failing treatments of infections due to the emergence of resistant bacterial pathogens has led to the awareness that this problem must be tackled from different angles, including a better understanding of the emergence and evolution of antibiotic resistance [2,10–12]. Resistance alleles can be acquired via horizontal gene transfer or *de novo* mutation, resulting in the resistant allele initially being present in one or a few individuals within a susceptible population. But how likely is it that this single genotype can establish in the population and what factors influence this process? Using time-lapse microscopy, Coates *et al*. [13] followed the growth of single *Escherichia coli* cells on agar, supplemented with different antibiotics. They found that the establishment of a single cell, determined by its growth into a visible macrocolony, was a highly stochastic process driven by fluctuations in cell death and birth, leading to the extinction of microcolonies at various time points. Similar results were found for two *Pseudomonas aeruginosa* strains, resistant to streptomycin or meropenem [14]. By seeding one to a few cells of a resistant strain in liquid medium with increasing antibiotic concentrations, it was found that at concentrations as low as one-eighth of the minimum inhibitory concentration (MIC) of this strain, its establishment probability was only 5% and that establishment of one cell was independent of the presence of other cells. The latter finding, however, may differ in systems where the antibiotic is broken down in the environment, leading to positive social interactions [15].

A common resistance mechanism potentially allowing for social effects involves β-lactamase enzymes. β-lactamases hydrolyse the lactam ring of β-lactam antibiotics, which target penicillin-binding proteins (PBPs) that are involved in cell wall synthesis of gram-negative bacteria, thereby leading to loss of cell wall integrity and eventual cell lysis [16,17]. A well-studied and clinically relevant example is TEM-1 β-lactamase, which has high activity towards first-generation penicillins, but low activity towards third-generation β-lactams, including the cephalosporin cefotaxime (CTX). However, the TEM-1 allele is the ancestor of a large family of extended-spectrum β-lactamases that have acquired mutations causing enhanced activity against more recently introduced penicillins and cephalosporins, including CTX [18–20]. As β-lactamases are expressed in the periplasmic space of gram-negative pathogens like *E. coli*, this system has been recognized as potentially cooperative, since the enzymatic breakdown in the periplasm reduces the antibiotic concentration in the environment [21], thereby cross-protecting susceptible bacteria nearby [15]. Such a cooperative behaviour can be expected to have more pronounced consequences on agar due to the local breakdown of the antibiotic, creating microenvironments that may allow for the coexistence of strains with different levels of antibiotic resistance [22–25].

Here, we examined the single-cell establishment probability of antibiotic-degrading bacteria under a range of antibiotic concentrations in both a structured (agar) and an unstructured (liquid) environment. We used four *E. coli* strains expressing β-lactamase alleles with different activities towards CTX, and hence different levels of resistance. Each strain was tested separately at low cell densities in both liquid and on agar medium with increasing CTX concentrations. To determine the single-cell establishment probability from the data, we developed a simple branching model, followed by a more detailed investigation of the cause of differences in stochasticity between the two environments. We show that environmental structure and cooperative behaviour play at most a minor role in the establishment of resistant cells at low initial densities. Based on our results, we further introduce a new measure for resistance level, MIC*_q_*(*N*), that takes inoculum size and stochasticity into account, thereby allowing for the detection of collective resistance. Application of the new measure to our data indicates substantial cooperative effects at higher cell densities. We therefore propose the new measure to be used for determining resistance levels as it overcomes the limitations of standard MIC assays.

## 2. Results and discussion

In this study, we aimed at quantifying how the establishment of single bacterial cells with different levels of antibiotic resistance is affected by: (i) the antibiotic concentration, (ii) the environmental structure and (iii) positive social interactions via the breakdown of the antibiotic in the environment. To determine single cell establishment probabilities, we performed one experiment in liquid (unstructured environment) and one on agar medium (structured environment). In each experiment, four *E. coli* strains were tested separately under exposure to a range of CTX concentrations: the TEM-1 Ancestor with very low β-lactamase activity, leaving this strain rather susceptible towards CTX, and three TEM mutant strains (referred to as Single mutant, Double mutant, Triple mutant; cf. table 1) with increasing levels of CTX resistance conferred by different antibiotic-hydrolysing β-lactamase enzymes. The tested concentrations differed between strains and were chosen to display relative establishment probabilities between 0 and 1 (i.e. relative to the absence of antibiotic; see Material and methods; electronic supplementary material, table S1). We use the notation *p*_e_(*x*) to denote the probability that a single cell establishes a macroscopic population (i.e. turbidity in liquid or colony formation on agar, visible to the naked eye) at CTX concentration *x*. When the antibiotic concentration is zero, a population should establish with maximum probability. Likewise, when the concentration is above the strains’ minimum inhibitory concentration (MIC), establishment is expected to be prevented. Strongly stochastic outcomes therefore arise only at intermediate concentrations.

**Table 1.** Overview of the used strains with their respective measured minimum inhibitory concentration (MIC) and null-model likelihood of the corresponding predicted MICs (see main text).

wild-type strain^a^ (without TEM)	TEM amino acid substitution	strain name (as used here)	MIC for Cefotaxime^b^ (µg ml^−1^)	null-model likelihood *L*^c^ (%)
MG1655 *galK*::SYFP2-CAT	none (TEM-1)	ancestor	0.08	>99.99
	G238S	single mutant	0.64	**1.1**
	E104 K/G238S	double mutant	10.24	**<0.01**
	E104 K/M182 T/G238S	triple mutant	81.92	**<0.01**

^a^MIC of the wild-type strain for CTX = 0.04 µg ml^−1^.

^b^As determined prior to the study by standard MIC assays (see Material and methods). Note that the true MIC lies between this value and half of this concentration (due to the 2-fold dilution during the setup).

^c^Assuming that the inoculum establishes at the highest concentration at which growth is observed in the MIC assay (see the last section of Results and Discussion); values that show significant differences between the measured and predicted MICs are depicted in bold.

### (a) Establishment probability declines well below the MIC in liquid

**Figure 1.**
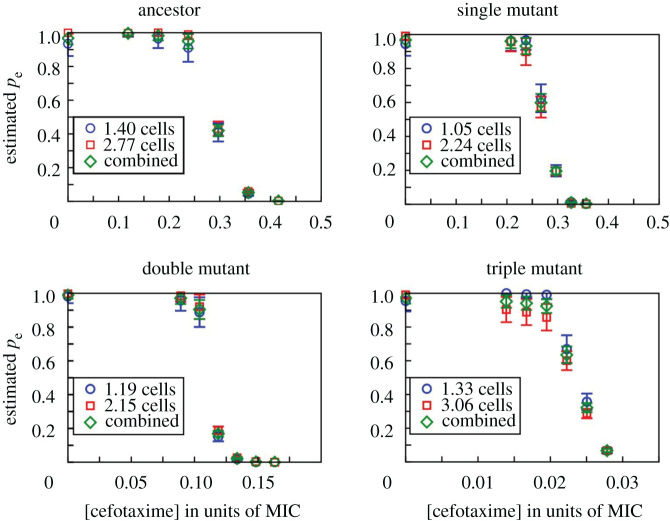
Single-cell establishment probabilities, *p*_e_, in liquid medium, estimated for the four tested strains as a function of antibiotic concentration, shown as a fraction of each strain's measured MIC (cf. table 1). For each strain, two inocula were used, one of approximately 1 cell (blue circles) and another of 2–3 cells (red squares); the average inoculum size estimated from separate CFU counts is shown. The inferred single-cell establishment probabilities are nearly identical for the two cases, implying that the establishment probability of a cell is not influenced by the presence of other cells at these low initial cell densities. The green diamonds are weighted averages over the two inoculum sizes (see electronic supplementary material, supplement S2 for details on the method used to estimate *p*_e_). (Online version in colour.)

In the liquid medium experiment, we grew bacteria in multi-well plates and observed their establishment as a function of CTX concentration. Per strain, inoculum size (about 1 and 3 cells on average) and antibiotic concentration, a total of 285 replicate wells were tested, spread across three plates, including one medium control well per plate. Estimates of the viable cell numbers present in the inocula for evaluating the probabilities were obtained by applying cell culture aliquots on agar, taken from the culture dilutions that were used for inoculating the well plates. Using a bootstrapping algorithm and assuming a Poisson distribution of independently acting cells in each inoculum (details in electronic supplementary material, S2), we estimated the single-cell establishment probability *p*_e_(*x*) for all strains and conditions. The results in figure 1 show that the establishment probability nears zero well below each strain's MIC and that this effect is more pronounced for strains with higher resistance levels.

The observation that the establishment probability of a single cell nears zero at antibiotic concentrations well below the strain's MIC has been shown before in *E. coli* for various bactericidal antibiotics [13] and in *P. aeruginosa* for streptomycin and meropenem [14]. These studies showed that the underlying causes for this are rooted in stochastic effects in population dynamics due to among-cell variability in death rate [13,14] and lag-phase [14], which become more pronounced with increasing antibiotic concentration. The establishment probability is higher with increasing initial cell numbers, simply because the probability is higher for at least one cell to survive and outgrow into a population [13,14]. This explains the decreased establishment probability of single cells at lower antibiotic concentrations compared to each strain's MIC, as the latter is measured for inocula containing hundreds of thousands of cells per ml. While we confirm the results of this general pattern in our study, we extend the findings by using four *E. coli* strains with the same genetic background but different sensitivities towards the β-lactam antibiotic CTX. As these differences in sensitivity are based on differences in the level of β-lactamase activity (i.e. enzymatic capacity in breaking down CTX), our system provides the opportunity to study the effect of positive social interactions during establishment. Indeed, the increasingly pronounced discrepancy between the single-cell establishment probability and standard MIC estimates that is observed with increasing resistance level is, at least partially, attributed to an increased collective breakdown at high cell densities (see section ‘MIC and the inoculum effect’ for details).

### (b) Cells do not interact in liquid medium during establishment

One complication in estimating the single-cell establishment probability in liquid medium is that multiple cells may be present initially in some wells, and these cells may in principle interact, for example through competition for resources or the breakdown of antibiotic molecules. In that case, the establishment probabilities of individual cells would no longer be independent, biasing our estimates of *p*_e_(*x*). To test whether cells interact within initially small inocula, we conducted the liquid experiment with two different mean inoculum sizes (with approximately 1 and 3 cells, respectively; see section 4(b)) and estimated *p*_e_(*x*) for both cases under the assumption that there is no interaction (details in electronic supplementary material, S2). If interactions occurred, the inferred values should disagree. However, figure 1 shows that they agree closely, indicating that interactions between cells are insignificant at these low cell densities in unstructured environments.

The absence of social interactions in liquid medium at low cell density was also found in the study with *P. aeruginosa*, exposed to streptomycin or meropenem under similar conditions regarding culture volume and inoculum size [14]. In that system, positive social interactions are less likely to occur, because streptomycin resistance does not involve antibiotic-degrading enzymes. Our *E. coli* β-lactamase system on the contrary involves the potential for cooperative effects due to an active breakdown of the antibiotic in the environment. The absence of an effect in our data confirms that cell-to-cell interactions (both positive and negative) play an insignificant role during the establishment of single cells at such low densities in liquid medium and further shows that this observation is independent of the resistance mechanism.

### (c) Establishment probability shows moderately higher stochasticity on agar

**Figure 2.**
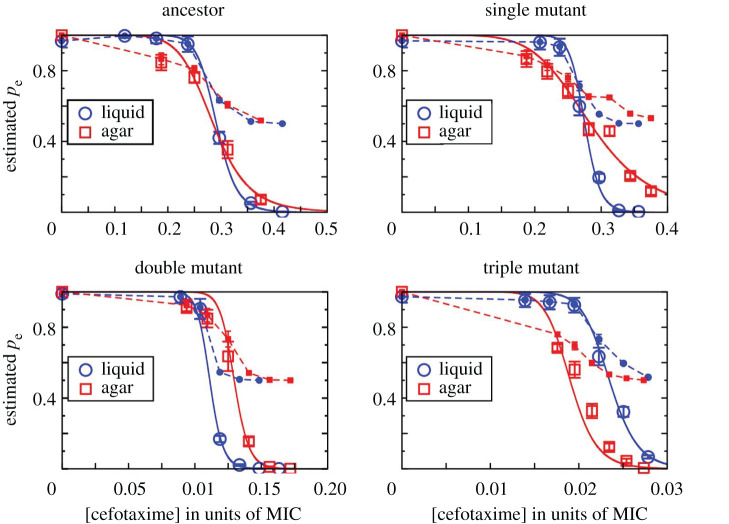
The single-cell establishment probability, *p*_e_, is shown as open symbols for the four tested strains in liquid (blue circles) and agar environments (red squares) for different CTX concentrations as the fraction of each strain's measured MIC. For liquid medium, the combined results from both tested inoculum sizes (cf. figure 1) are shown. The solid lines are fits performed with the Hill function *p*_e_(*x*) = (1 + (*x*/*x*_0_)*^n^*)^−1^ with *x*_0_ and *n* as parameters, where *x*_0_ is the concentration at which *p*_e_ drops to 1/2, and *n* indicates the steepness of the curve. The values of the inferred parameters are given in electronic supplementary material, table S2. The filled symbols connected by dashed lines show the cell division probability *p_d_* inferred from a simple branching model for the data points where *p*_e_ > 0 (see main text). (Online version in colour.)

In the previous section, we found that the establishment probability of one cell was not influenced by the presence of other cells in liquid medium at very low densities. But how is this on agar? Previous studies have shown that evolutionary outcomes can largely differ depending on the environmental structure in the absence of migration [26–28]. For instance, while conditions in unstructured (liquid) environments commonly select for one strain to dominate the population, structured (agar) environments can maintain various strains due to the creation of microenvironments [29–31]. One condition that can be affected locally on agar is the concentration of the antibiotic due to enzymatic breakdown. While it has been shown that this can result in cooperative effects, where a resistant strain protects more sensitive ones [15,32] and such cooperation is more prominent in structured environments [25], it is, to the best of our knowledge, unknown whether such social interactions can influence the establishment of single antibiotic-resistant cells. To determine the effect of environmental structure on the establishment of β-lactamase-expressing cells, we plated about 200 cells of each *E. coli* strain on CTX-containing agar (eight plates per condition), followed by counts of colony-forming units (CFUs) after incubation. As for the experiment in liquid medium, the tested CTX concentration range depended on the strain to display establishment probabilities between 0 and 1 (see Material and methods; electronic supplementary material, table S1). The establishment probability was estimated as the CFU count relative to that at no CTX. The patterns of *p*_e_ as a function of CTX concentration are broadly similar to those in liquid medium (figure 2), indicating a minor role of environmental structure being involved in mutant establishment at low cell densities.

**Figure 3.**
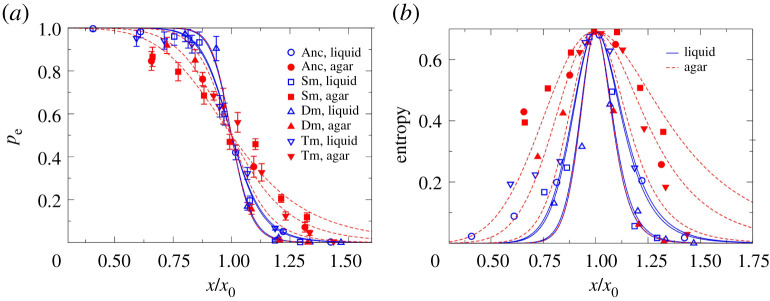
(*a*) Establishment probabilities for all the strains in both liquid and agar environments as a function of CTX concentration (Anc: Ancestor, Sm: Single mutant, Dm: Double mutant, Tm: Triple mutant), scaled by the corresponding estimate *x*_0_ (i.e. the concentration at which *p*_e_ drops to 0.5). The curves for agar medium (dashed red lines) are less steep than those for liquid medium (blue solid lines). (*b*) The Shannon entropy is calculated and plotted for the *p*_e_ values, using the standard expression *S* = −*p*_e_ln*p*_e_ − (1 − *p*_e_)ln(1 − *p*_e_). It is seen to be higher for agar (dashed red lines and filled symbols) compared to liquid (solid blue lines and open symbols), as expected from the shallower slopes of the *p*_e_(*x*) curves for agar in panel (*a*). (Online version in colour.)

Although we found a general agreement of the establishment probability pattern between the liquid and agar experimental data, a closer comparison of the estimates of both environments shows that the decline of *p*_e_ with CTX concentration is less steep on agar than in liquid (figure 3*a*). This implies a higher degree of stochasticity on agar, since *p*_e_ remains between 0 and 1 over a larger range of concentrations. We quantified this effect by calculating the Shannon entropy for the establishment probabilities in both environments and all four strains. Shannon entropy is an information-theoretic measure that indicates the degree of stochasticity associated with a set of probabilities. In our context, the measure has a value of 0 (no stochasticity) in the extreme cases of *p*_e_ = 0 or *p*_e_ = 1, and is maximized at the intermediate value of *p*_e_ = 0.5. Figure 3*b* shows our finding that the entropy, and therefore the stochasticity associated with the outcome of the establishment process, is higher on agar.

Next, using a modelling approach we explored possible underlying mechanisms for stochasticity in the system. The simplest model is a branching process, where every cell divides with a fixed probability *p_d_*, or dies otherwise; the population either continues to grow indefinitely (with probability *p*_e_) or eventually goes extinct (with probability 1 − *p*_e_; see electronic supplementary material, S3 for details). This model allows us to estimate the division probability *p_d_* using the measured *p*_e_ values. Figure 2 shows that under the simple branching model, the division probability appears to approach the value ½ asymptotically as opposed to the natural expectation of approaching zero asymptotically (see electronic supplementary material, figure S1 for an illustration of the possible shapes of the *p*_e_ and *p_d_* curves in the simple branching model). This is a clear indication that the simple model does not fully capture the establishment process. One way to modify the model is to introduce variability in *p_d_* among cell lineages, and this fits the data reasonably well (see electronic supplementary material, S4). Within this framework, the higher establishment stochasticity on agar could be due to small differences in *p_d_* of different cells on the agar surface (see electronic supplementary material, S4 and figure S3 for details). However, other possibilities remain; for instance, the cell division probability may change with time due to accumulating damage in cell walls under antibiotic action, changing rates of antibiotic and enzyme flux or changing CTX concentration over time at the location of a growing colony. Distinguishing conclusively between these alternatives is not possible based on our data, and we do not explore this issue any further here.

### (d) MIC and the inoculum effect

Results from our experiments in liquid medium have shown a discrepancy between the CTX concentration where the single-cell establishment probability, *p*_e_, nears zero and the standard MIC value. As mentioned above, the reasons for this lie in the fact that standard MIC values are measured from large inocula of 5 × 10^5^ cells ml^−1^, while establishment is a highly stochastic process at the cellular level. The stochastic nature of MIC estimates can be seen from the limited reproducibility of MIC assays, despite their coarse scale [13].

A conceptually cleaner approach to determine the MIC of a strain would therefore be to focus on a quantity that considers the role of stochasticity and initial inoculum size in the establishment of a population. For this purpose, we define the measure MIC*_q_*(*N*) as the minimum concentration at which an inoculum of initially *N* cells has a probability *q* of not being established (see electronic supplementary material, S6). We suggest a null-model for this quantity, which assumes that the fates of the lineages arising from the *N* initial cells are independent, and therefore the extinction probability of the entire population equals (1 − *p*_e_)*^N^*. Predictions based on this null-model can be used to test for the presence of interactions in the establishment process. For example, in a traditional MIC assay with an inoculum of 10^5^ cells/200 µl, the null model predicts that ${\mathrm{MIC}}_{\frac{1}{2}}(10^{5})$ has the values of 0.053 (±3.5%), 0.31 (±4.7%), 2.0 (±3.6%) and 4.5 (±6.6%) µg ml^−1^ for the Ancestor, Single mutant, Double mutant and Triple mutant, respectively (see Supplement S6 for further details). The differences between these predicted values and the experimentally determined MICs (cf. table 1) become increasingly pronounced with the level of resistance of the mutants. A choice of *q* other than 0.5 produces only modest changes in the predicted values (unless *q* is very close to the limits 0 or 1; see electronic supplementary material, S6), and given the limited resolution of the MIC assay with two-fold increases in concentration, the precise choice of *q* does not affect the conclusion. The pattern of differences between the null-model MIC_1/2_ prediction and the measured MICs prompted us to calculate the likelihood, *L*, based on the null-model, that the inoculum establishes at the highest concentration at which growth is observed in the MIC assay (i.e. at half the reported MIC, due to the two-fold dilution steps). The results are shown in table 1. While *L* is high for the Ancestor, it is low for all the mutants (*L* < 2%), indicating that the measured MIC for the mutants is significantly above what we predict in the absence of social interactions (table 1). This suggests that positive interactions among cells present at high density substantially enhance the collective establishment of mutant cell populations.

The effect of inoculum size on the MIC through social interactions has been recognized before [33,34] and causes have been suggested to include the binding of the antibiotic to cellular components [35] or the expression of proteins that inactivate the antibiotic [22,36]. The four *E. coli* strains used here are genetically identical apart from the point mutations in the TEM allele, meaning that the number of penicillin-binding-proteins (PBPs), which are the main binding target of CTX [37,38], are comparable between them. Thus, binding of CTX molecules, although possibly contributing to the discrepancy between measured and predicted MICs, cannot explain the increasingly pronounced differences between the two resistance measures with increasing resistance levels of the mutant strains. As the level of resistance is conferred by increased enzyme activity against CTX with each additional point mutation [39], whereas the β-lactamase activity of the Ancestor is very low, the collective breakdown of the antibiotic in the classical MIC assay is a likely explanation for the observed effect. Overall, our results on the discrepancy between the standard MIC value and the single-cell establishment probability are in line with the findings of other studies that criticized the MIC assay for dosage determination for the treatment of antibiotic-resistant infections [14,40,41].

## 3. Conclusion

This study advances our understanding of the stochastic nature of the establishment of initially rare adaptive mutants (e.g. due to *de novo* mutation), a topic that has been under much theoretical scrutiny, but has rarely been investigated empirically. We find that the establishment of *E. coli* single mutant cells is negatively affected by CTX concentrations well below each of the four tested strain's MIC, consistent with other recent findings [14].

One aim of our study was to test if cooperative behaviour via collective antibiotic breakdown among cells of the same strain, would affect the establishment probability and whether this would differ between unstructured and structured environments. Despite higher stochasticity on agar, our data show a reasonable agreement between the establishment in liquid and agar environments, indicating that environmental structure plays only a modest role, and that cooperative behaviour has a negligible effect on the establishment probability of single resistant mutant cells in isolation. However, the process of establishment of antibiotic resistant mutants is particularly relevant for *de novo* mutants arising in populations of relatively susceptible bacteria, and in recent work, we demonstrated positive net effects on mutant establishment from susceptible cells in the same experimental system [42].

Lastly, we point out limitations of the traditional MIC measurements that make MIC values highly sensitive to the assay conditions. We suggest a more robust measure of resistance, MIC*_q_*(*N*), which includes both inoculum size and probabilistic effects, for which we constructed a null model based on the single-cell establishment probability, *p*_e_, by assuming homogeneous cell populations and no social interactions across lineages. The null-model prediction disagrees with the measured MIC in systematic ways, indicating strong cooperative effects at high cell densities [42]. We propose that this measure may be used as a reference for detecting conditions of collective resistance for other bacterial strains and antibiotics.

## 4. Material and methods

### (a) Strains and culture conditions

Four strains were used for the experiments, derived from *Escherichia coli* strain MG1655 *galK*::SYFP2-CAT (a kind gift from the laboratory of Dan Andersson via Peter Lind). This strain (DA28100) had previously been modified with a YFP (yellow fluorescent protein) marker cassette, containing a resistance gene for chloramphenicol [43]. For the current study, the chloramphenicol resistance was removed, and each one of four TEM variants was inserted into the *galK* locus, together with the pTac promoter from plasmid pACTEM [44]: the ancestral TEM-1 allele (referred to as ‘Ancestor’), and three mutant alleles with either 1, 2 or 3 point mutations in the TEM allele (referred to as ‘Single mutant’, ‘Double mutant’ and ‘Triple mutant’, respectively). While the ancestral TEM-1 allele confers only low activity towards the β-lactam antibiotic cefotaxime (CTX), the three mutants show increasing CTX resistance with each additional substitution (table 1). All TEM loci are under the control of the LacI repressor and are expressed by adding 50 µM Isopropyl β-D-1-thiogalactopyranoside (IPTG) to the growth medium. The minimum inhibitory concentration (MIC) of each strain for CTX had previously been determined in duplicates (table 1), using 2-fold increases in CTX concentration in microtiter plates filled with 200 µl M9 minimal medium (containing 0.4% glucose, 0.2% casaminoacids, 2 µg ml^−1^ uracil and 1 µg ml^−1^ thiamine) and 50 µM IPTG, inoculated with 10^5^ cells and incubated for 24 h at 37°C.

For culturing, all strains were first recovered from glycerol stocks by streaking them out on LB (Luria-Bertani) agar plates and incubated overnight at 37°C. From those plates, one colony was picked and introduced into 1 ml M9 medium (as above) and incubated overnight at 37°C, 250 rpm. The cultures were then serially diluted with phosphate-buffered saline (PBS) to the density needed for the particular experiment (see below), assuming an initial density of 2.5 × 10^9^ cells ml^−1^.

### (b) Liquid experiment

Each of the four strains was tested separately in the absence of CTX and six CTX concentrations (electronic supplementary material, table S1), where the particular tested concentrations depended on the strains' resistance level. The range of concentrations was chosen to display establishment probabilities between 0 and 1, based on pilot experiments to find the right conditions (data not shown).

Per strain and CTX concentration, on average either about 1 or 2–3 cells were seeded into 285 wells across three 96-well microtiter plates. For this, 190 µl M9 medium with the respective cefotaxime concentration and 50 µM IPTG were pipetted into all wells. Overnight cultures of the strains were diluted to 200 and 100 cells ml^−1^ with PBS. 10 µl of the dilutions were pipetted into the wells. One well per plate served as medium control, mock-inoculated with 10 µl PBS. The plates were incubated for about 40 h at 37°C (static). After incubation, OD at 600 nm of all wells was measured in a plate reader (Victor3, PerkinElmer) without the plate lid. All medium controls across the experiment showed no sign of growth; the OD ranged from 0.033 to 0.038. Thus, a threshold of greater than 0.05 was applied to determine whether a population has been established successfully.

To determine the mean number of seeded cells in culture dilutions, aliquots of the dilutions were dropped on LB agar plates (120 × 120 mm). Per dilution and strain, a total of 96 10 µl drops were applied across 3 plates (32 drops each), let dry for about 10 to 20 min, incubated at 37°C for about 2–3 h until leaving, and then moved to 30°C overnight to prevent overgrowth, followed by counting the number of colonies per droplet.

### (c) Agar experiment

As in the liquid experiment, all strains were tested separately and the CTX concentration range depended on the strain. Five to eight concentrations (including a 0 CTX treatment to estimate the relative CFU count) were tested per strain (electronic supplementary material, Supplement S1, table S1). The overnight cultures were diluted to 4000 cells ml^−1^. 50 µl (approx. 200 cells) of the dilutions were spread onto 92 mm agar plates containing M9 medium (as above) with 1.5% agar, 50 µM IPTG and the respective CTX concentration, using a bacterial spreader. Per strain and cefotaxime treatment, eight replicate plates were used. The plates were incubated at 37°C until colonies were big enough to count them unambiguously (20 to 48 h). Each colony is regarded as a successfully established single cell.

## Data Availability

Supplementary information is provided in electronic supplementary material [45].
